# Variation in nuclear genome size within the Eisenia nordenskioldi complex (Lumbricidae, Annelida)

**DOI:** 10.18699/VJ21.073

**Published:** 2021-10

**Authors:** S.V. Shekhovtsov, Ya.R. Efremov, T.V. Poluboyarova, S.E. Peltek1

**Affiliations:** Kurchatov Genomic Center of ICG SB RAS, Novosibirsk, Russia; Institute of Biological Problems of the North of the Far-Eastern Branch of the Russian Academy of Sciences, Magadan, Russia; Kurchatov Genomic Center of ICG SB RAS, Novosibirsk, Russia; Kurchatov Genomic Center of ICG SB RAS, Novosibirsk, Russia; Kurchatov Genomic Center of ICG SB RAS, Novosibirsk, Russia

**Keywords:** earthworms, Eisenia nordenskioldi, genome size, flow cytometry, phylogeny, дождевые черви, Eisenia nordenskioldi, размер генома, проточная цитофотометрия, филогения

## Abstract

The size of the nuclear genome in eukaryotes is mostly determined by mobile elements and noncoding sequences and may vary within wide limits. It can differ signif icantly both among higher-order taxa and closely related species within a genus; genome size is known to be uncorrelated with organism complexity (the so-called C-paradox). Less is known about intraspecif ic variation of this parameter. Typically, genome size is stable within a species, and the known exceptions turn out be cryptic taxa. The Eisenia nordenskioldi complex encompasses several closely related earthworm species. They are widely distributed in the Urals, Siberia, and the Russian Far East, as well as adjacent regions. This complex is characterized by signif icant morphological, chromosomal, ecological, and genetic variation. The aim of our study was to estimate the nuclear genome size in several genetic lineages of the E. nordenskioldi complex using f low cytometry. The genome size in different genetic lineages differed strongly, which supports the hypothesis that they are separate species. We found two groups of lineages, with small (250–500 Mbp) and large (2300–3500 Mbp) genomes. Moreover, different populations within one lineage also demonstrated variation in genome size (15–25 %). We compared the obtained data to phylogenetic trees based on transcriptome data. Genome size in ancestral population was more likely to be big. It increased or decreased independently in different lineages, and these processes could be associated with changes in genome size and/or transition to endogeic lifestyle.

## Abstract (ru)

Размеры ядерного генома у большинства эукариот определяются преимущественно содержа- нием мобильных элементов и некодирующих последовательностей и варьируют в широких пределах. Они могут значительно различаться как между крупными таксонами (причем размер генома не коррелирует со сложностью организма – так называемый С-парадокс), так и между близкородственными видами в преде- лах рода. В то же время размах внутривидовой изменчивости по этому параметру изучен значительно хуже. Комплекс Eisenia nordenskioldi объединяет несколько близкородственных видов дождевых червей, широко распространенных на Урале, в Сибири и на Дальнем Востоке России, заходящих своими ареалами и в сопре- дельные регионы. Для этого комплекса характерна значительная морфологическая, кариотипическая, эко- логическая и генетическая изменчивость. Целью настоящей работы было оценить размеры ядерного генома у нескольких филогенетических линий комплекса E. nordenskioldi при помощи проточной цитофотометрии. Получены данные о размерах генома для 13 популяций, относящихся к семи филогенетическим линиям E. nordenskioldi. Наши результаты показали, что между линиями комплекса наблюдается заметный разброс по размерам, что является еще одним подтверждением их видовой самостоятельности. В целом по разме- ру генома выборки разделены на две группы. В одну вошли три популяции с небольшим (250–500 м. п. н.), во вторую – с крупным (2300–3500 м. п. н.) размером генома. Кроме того, разные популяции в пределах одной филогенетической линии также имели заметные различия в размере генома (15–25 %). Полученные данные были сопоставлены с филогенетическими деревьями, построенными на основе транскриптомных данных Судя по топологии филогенетических деревьев, предковые популяции комплекса с большей вероятностью имели большой размер генома, а уменьшение или увеличение его размера происходило в разных линиях независимо и, возможно, было связано с изменением размеров тела и/или переходом к собственно почвен- ному образу жизни.

## Introduction

The amount of nuclear DNA in eukaryotes varies widely and does not correlate with the complexity of an organism (Cavalier-Smith, 1978; Gregory, 2001). This phenomenon was dubbed the “C-paradox” (Thomas, 1971). Patterns of genome size variation are currently well studied both for higher-level taxa and for groups of closely related species from many diverse phyla (Gregory, 2005). The patterns of intraspecific diversity are generally less known. It is generally believed that genome size and architecture must be common in different populations so they remain genetically and reproductively compatible, i. e. remain a species. There are certain deviations from this rule: differences between males and females due to sex chromosomes; the presence of additional B-chromosomes or large blocks of heterochromatin (Gregory, 2005; Biémont, 2008). However, in most cases intraspecific diversity does not exceed several percent (Blommaert, 2020). The known cases of high variation in intraspecific genome size (Alvarez-Fuster et al., 1991; Marescalchi et al., 1998; Neiman et al., 2011; Stelzer et al., 2011; Jeffery et al., 2016) are often explained by the presence of the so-called cryptic, or sister, species, which were not detected earlier.

The Eisenia nordenskioldi (Eisen, 1874) complex is a group of species/genetic lineages of earthworms from the Lumbricidae family widespread in Asian Russia and also found in the East European Plain and certain adjacent countries (Perel, 1979; Zhukov et al., 2007; Blakemore, 2013; Hong, Csuzdi, 2016; Shekhovtsov et al., 2017b). This complex is known for its enormous morphological (Malevich, 1956; Perel, 1979; Vsevolodova-Perel, 1997), karyotypic (Graphodatsky et al., 1982; Vsevolodova-Perel, Bulatova, 2008), ecological (Berman et al., 2019), and genetic (Malinina, Perel, 1984; Shekhovtsov et al., 2013, 2016a, b, 2017a, 2018a, b) diversity. Phylogenetic studies using genomic and transcriptomic data confirmed deep divergence between the lineages of this complex (Shekhovtsov et al., 2019, 2020a, b) and suggested that it could be divided in at least two distinct species.

Remarkable differences between the nuclear and mitochondrial genomes of E. nordenskioldi genetic lineages indicate that they diverged long ago (Shekhovtsov et al., 2013, 2015). Significant variation in genome size not associated with polyploidy could thus have accumulated in this complex. To elucidate this question we studied genome size in several genetic lineages of E. nordenskioldi using flow cytometry.

## Materials and methods

Live earthworms were collected in 2020 in various locations from the Urals, Siberia, and the Far East (see the Table). The warms were rinsed, placed individually in Petri dishes with wet paper and kept for 3–7 days. Genome size was estimated according to the fluorescence of DAPI-stained nuclei of individual cells according to the technique of D.W. Galbraith et al. (1997). Nuclei were isolated either from several posterior segments of a live earthworm (100–300 μg) or from the whole animal if it was small. A part of the material (about 50–100 μg) was fixed in ethanol for DNA extraction as described below.

Live material was placed in a Petri dish with 500 μl of Galbraith buffer: 45 mM MgCl2, 20 mM 3-[N-morpholino] propanesulfonic acid (MOPS), 30 mM sodium citrate, 0.1 % Triton X-100 (Galbraith et al., 1983). Material was grinded by multiple strokes with a razor blade. Liquid phase was transferred into an Eppendorf tube. Another 500 μl of Galbraith buffer was added to the Petri dish, and liquid phase was again transferred to the Eppendorf tube. The sample was incubated for 15–60 min, filtered through a 40 μm mesh, and placed on top of 2 ml Galbraith buffer with 3 % glycerol. The tube was centrifuged for 10 min at 200 g; supernatant was discarded, the sediment was dissolved in 500 μl of Galbraith buffer with 10 μl RNAse (1 u/μl). The sample was incubated for 30 min, mixed with 100 μl of propidium iodide (1 mg/ml) and analyzed on a FACSAria III flow cytometer (BD Biosciences, USA). We used chicken blood cells (2C = 1250 Mbp) (Kasai et al., 2012) and mouse spleen cells (2C = 3280 Mbp) (Redi et al., 2005) as the reference.

To determine genetic lineage, we sequenced a fragment of the mitochondrial cytochrome oxidase I gene as described in (Shekhovtsov et al., 2018c). Phylogenetic trees built using the Maximum Likelihood and Bayesian inference algorithms were taken from S.V. Shekhovtsov et al. (2020b).

## Results and discussion

In this study we determined genome size for several genetic lineages of the E. nordenskioldi complex (see the Table and Figure). The obtained data indicate high variation in genome size in this complex. We could distinguish two size classes: small (250–500 Mbp) and large (2350–3500 Mbp) genomes. Small genomes were observed in three cases, for two nonpigmented lineages of Eisenia sp. 1 aff. E. nordenskioldi and for the pigmented lineage 2 of this species. Large genomes (2350–3500 Mbp) were found in the rest of the lineages.

**Tab:**
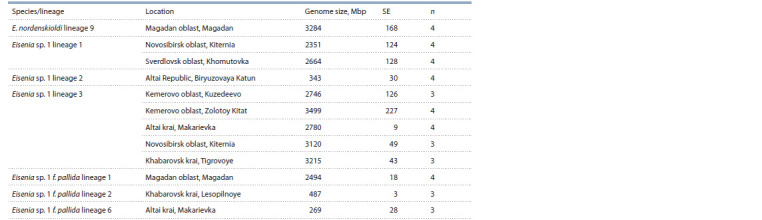
Studied specimens Notе. SE – standard error; n – number of individuals.

**Fig.**
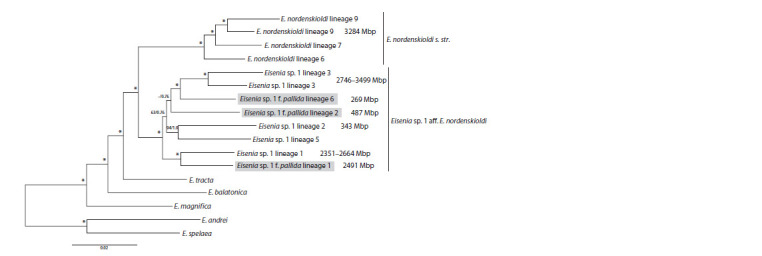
Phylogenetic tree constructed for the E. nordenskioldi complex based on transcriptomic data, taken from (Shekhovtsov et al., 2020b). Grey squares denote the non-pigmented pallida form. Numbers near the branches indicate Maximum Likelihood bootstrap support/Bayesian posterior probabilities; asterisks stand for 100/1.0.

Thus, different genetic lineages of the E. nordenskioldi have strongly diverged genomes. This could imply that these lineages represent distinct species (Shekhovtsov et al., 2020a, b), or that this is the result of polyploidy in this complex. It is known that E. nordenskioldi consists of races with different ploidy: 2n, 4n, 6n, 7n, 8n, with the chromosome number ranging from 36 to 142–152 (Viktorov, 1997). Diploid chromosome set is believed to be characteristic for the non-pigmented pallida form (Viktorov, 1997; Vsevolodova-Perel, Leirikh, 2014). Based on this, it would be reasonable to suggest that the diploid non-pigmented forms are ancestral to this complex. However, transcriptomic data demonstrated (see the Figure) (Shekhovtsov et al., 2020b) that these forms are not at the basis of the tree, and the ancestral forms were pigmented.

Moreover, one of the pallida lineages had a large genome while one pigmented lineage had a small one. Therefore, one cannot state that all non-pigmented forms are diploid and pigmented ones are always polyploid. Moreover, the pallida form arose independently several times

The same arguments apply to genome size: it seems more probable that the ancestral genome was large. Moreover, since the majority of E. nordenskioldi populations are amphimictic, the ancestor of the complex was amphimictic and diploid. For Eisenia sp. 1, the tree topology also implies that large nuclear genome was the ancestral state, and some branches (lineages) subsequently went through genome compaction.

Several populations from diverse geographic locations were sampled for two genetic lineages (lineages 1 and 3 of Eisenia sp. 1). Our analysis demonstrated that there is a certain genome size diversity within these lineages, approximately 13 and 27 % for lineages 1 and 3, respectively. It is well known (Viktorov, 1997; Vsevolodova-Perel, Bulatova, 2008) that chromosome number in octaploid E. nordenskioldi populations varies widely, and we may suggest a similar mechanism in this case.

Polyploidy results in increased body size in many animals (Otto, 2007). Earthworms, however, may not conform to this pattern: T.V. Malinina and T.S. Perel (1984) found no size differences between E. nordenskioldi of different ploidy. Here we could not measure body size, because the studied animals were completely or partially grinded. However, rough estimates suggest that genetic lineages with small genomes were small or average in size (4–7 cm long), while those with large genomes could be either large (to over 10 cm for Eisenia sp. 1 lineage 3) or average (5–10 cm for other lineages). Therefore, although we did not observe a clear pattern, we could hypothesize that genome size partially accounts for body size.

## Conclusion

In this study we demonstrated that nuclear genome size varies widely among genetic lineages of the E. nordenskioldi complex. This corroborates the remarkable differences among them demonstrated by molecular genetic methods. Moreover, there was also some variation between different populations of the same lineage. Both genome expansion and contraction occurred during the evolution of the complex.

## Conflict of interest

The authors declare no conflict of interest.
